# Digital Images Authentication Technique Based on DWT, DCT and Local Binary Patterns

**DOI:** 10.3390/s18103372

**Published:** 2018-10-09

**Authors:** Esteban Alejandro Armas Vega, Ana Lucila Sandoval Orozco, Luis Javier García Villalba, Julio Hernandez-Castro

**Affiliations:** 1 Group of Analysis, Security and Systems (GASS), Department of Software Engineering and Artificial Intelligence (DISIA), Faculty of Computer Science and Engineering, Office 431, Universidad Complutense de Madrid (UCM), Calle Profesor José García Santesmases, 9, Ciudad Universitaria, 28040 Madrid, Spain; esarmas@ucm.es (E.A.A.V.); asandoval@fdi.ucm.es (A.L.S.O.); 2 School of Computing, Office S129A, University of Kent, Cornwallis South Building, Canterbury CT2 7NF, UK; J.C.Hernandez-Castro@kent.ac.uk

**Keywords:** digital images, discrete cosine transforms, forgery detection, image forensics, images splicing, local pattern binary, support vector machines, wavelet transforms

## Abstract

In the last few years, the world has witnessed a ground-breaking growth in the use of digital images and their applications in the modern society. In addition, image editing applications have downplayed the modification of digital photos and this compromises the authenticity and veracity of a digital image. These applications allow for tampering the content of the image without leaving visible traces. In addition to this, the easiness of distributing information through the Internet has caused society to accept everything it sees as true without questioning its integrity. This paper proposes a digital image authentication technique that combines the analysis of local texture patterns with the discrete wavelet transform and the discrete cosine transform to extract features from each of the blocks of an image. Subsequently, it uses a vector support machine to create a model that allows verification of the authenticity of the image. Experiments were performed with falsified images from public databases widely used in the literature that demonstrate the efficiency of the proposed method.

## 1. Introduction

In recent years, the use of mobile devices has increased considerably, becoming a tool that is part of the daily life of today’s society. In 2017, a report by Cisco Systems [1] shows that the mobile data traffic has multiplied by 18 in the last five years and this traffic is expected to continue increasing. This information also was confirmed in 2018 by Ericsson in its report [2] in which indicates that by the year 2023 the mobile data traffic will be multiplied by 7 and almost three-quarters of the world’s mobile data traffic will be used for multimedia and social media networks.

The multimedia content (images and video) are, thanks to the social media networks and instant messaging applications, most data traffic consumers. Given the powerful and omnipresent nature of digital media, specifically digital images, it is reasonable that the awareness of image forgery and its detection gain are of significant importance.

On the other hand, the continuous improvement of the cameras incorporated in mobile devices together with the evolution of the image editing tools have made it easier to manipulate an image with excellent results. To deal with this massive traffic of possible manipulated images, the forensic analysis area investigates new manipulation detection techniques to evaluate the integrity of an image. Manipulated images have been around for decades and are present in many sectors (politics, cinema, press, judicial branch, etc.). Detect manipulated digital images are of great importance in many areas and with different objectives. As it is the case in the legal sector, where the images or videos can suppose evidence of great value for the resolution of the lawsuit. An example of this was the arrest of a driver who was driving his car at more than 200 Km/h and the evidence was the video recorded by a pedestrian, where the driver was shown circulating at that speed and immediately was reported him to the police [3]. However, in order for an image to be used as valid evidence in a trial, its integrity must be assured and demonstrated that it has not been manipulated. In order to carry out this type of authentication, it is necessary to make use of robust manipulation identification techniques that can assure with high security that the image is original. For all of the above, it is necessary to study and develop forensic techniques to identify any amount of manipulated images that can be found in these days.

The rest of the work is organised as follows: Section 2 details the types of techniques and tools used in the manipulation of digital images. Section 3 describes the main techniques of manipulated image detection, highlighting the most relevant passive approach techniques in the literature. The details of the proposed detection technique are presented in Section 4. Section 5 analyses the results of the experiments carried out and, finally, the conclusions of the work are included in Section 6.

## 2. Related Work

There are several forensic approaches to image manipulation detection and, commonly, these approaches could be divided into: intrusive or active and non-intrusive or passive approaches [4].

- **Active Approach**: This approach analyzes the watermarks or signals left by a device when generating a digital image. Authenticity is checked by detecting changes in these watermarks. The biggest drawback of this type of approach is that many cameras don’t have the capacity to incorporate these types of marks or signatures, so their scope is limited.
- **Passive Approach**: This approach analyzes the content and characteristics of the digital image. Moreover, this approach can be classified as learning-based methods and block-based methods.
  – **Learning-based Methods**: These methods have the ability to detect any type of forgery, determine whether an image has been manipulated or not based on a previous training. In social media networks where it is sufficient to verify if an image is original or manipulated, learning-based methods are used due to its great capacity to classify large datasets at a reduced cost of time and resources.
  – **Block-based Methods**: These methods detect falsification by locating the regions that have been adulterated (e.g., copy/paste forgery). Block-based methods are useful for presenting evidence in courtrooms or insurance claims. Their biggest disadvantage is the consumption of time and resources, which makes them unsuitable for applications such as social networks, where a large amount of images is shared every day.

The passive approach has an ubiquitous scope than the active approach since it does not need prior information about the images. The most relevant passive approach proposals are presented below.

In [5], Kee et al. proposed an algorithm designed to predict the presence of retouching in magazine cover images. The dataset of 468 photos (original and retouched) was evaluated with values between 1 (very similar) and 5 (very different) depending on the amount of image alteration. The geometrical and photometric modifications of each original and retouched photo were calculated and, later, eight summary statistics were extracted that incorporate the degree of photographic retouching to calculate the correlation with the evaluation of each photo. A Support Vector Machine (SVM) algorithm was used to determine the degree of modification of the image. The maximum precision obtained with the experiments was 98.75%.

The detection of retouching in images can also be applied to social media networks. In these applications, most user profile images show mainly its face and some of these users apply retouching techniques to prevent their identification, showing a face that does not correspond to its own. This is another reason to develop strong algorithms that allow for determining a possible manipulation in any part of the image, despite how small it may be.

The algorithm proposed by Bharati et al. in [6] uses a neural network to extract features and SVM to classify the images in an *unretouched* or *retouched* class. In the experiments, the used dataset was “*ND-IIITD Retouched Faces*” which contain 325 retouched faces. The obtained results show an 87% of success.

An important problem related to facial images is virtual makeup. This specific type of retouching generates a simulation of real make-up on one face, which can hinder the process of facial identification of a person. For the automatic detection of makeup, several methods have been proposed.

In [7], Chen et al. propose the extraction of the characteristics of color, shape, and texture of three predefined facial regions of the image. In the experiments, the datasets YMU and MIW [8] were used to train and predict, respectively, a system SVM with Radial Basis Function (RBF) kernel to classify them. The precision obtained was 93%. Later, in [9], Kose et al. proposed a more precise algorithm for the detection of makeup in the same datasets using texture and shape characteristics. The proposed technique extracts a feature vector that captures the shape and texture characteristics of the face used as input to the algorithm. The accuracy was increased to 98.5% using an SVM classifier.

The forgery of splicing of images is common and relatively easy to perform. In the current digital age, this kind of manipulation can be executed without leaving visible traces and the post-processing tasks make it more difficult to detect. In summary, splice detection can be considered as a problem of detecting a weak signal (splicing artifact) at the bottom of a strong signal (image content).

The splice detection method proposed in [10] by Shi et al. models the manipulation changes using statistical characteristics extracted from 2D matrices generated by applying multi-size block discrete cosine transform (MBDCT). In the experiments, a 91.40% accuracy was obtained on the dataset “*Columbia*” [11] using SVM.

In [12] the authors Z. Zhang et al. represent alteration changes using characteristics extracted from 2D matrices generated when applying MBDCT and image quality metrics (IQM) and also using SVM. The “*Columbia*” dataset is used and it obtains a precision of 87.10%.

In [13], the authors Y. Zhang et al. apply local binary pattern (LBP) to extract 2D arrays of image features after the application of multi-size block discrete cosine transform (MBDCT), the main component analysis; PCA was also used to reduce the dimensionality. Finally, with the use of a support vector machine (SVM), the classification was made. After all this, the results obtained were of a precision of 89.93 % using “*Columbia* ” dataset.

Wang et al. in [14] propose a method to detect splicing image forgery based on modeling edge information. The edge information is extracted from the chromatic component using a finite-state Markov chain and SVM as a classifier. The results of the experiments carried out obtained an accuracy of 95.6 % with the dataset “*CASIA TIDE v2.0*” [15].

In [16], Zhao et al. explored the effect of different color models on the detection of splicing forgery. In this work, a comparison of the chromatic models is made against the commonly used Red, Green, Blue (RGB) and luminance models. Four gray level Run-Length Run-Number (RLRN) vectors with different directions extracted from correlated chrominance channels are used as characteristics for the detection of splicing in images. Finally, a support vector machine SVM is used as a classifying algorithm. This research revealed that chrominance channels are more effective in detecting counterfeits. The dataset used in the experiments was “*CASIA TIDE v1.0*” and “*Columbia*” with an accuracy result of 94.7%.

The authors Xia et al. in [17], present a method to detect falsification in fingerprints images, the method that uses discrete wavelet transform (DWT), employed with local binary patterns (LBP), manages to obtain an accuracy of 92%. Different images of fingerprints obtained by different types of scanners were used for the experiments. All of these images were obtained from the dataset “*LivDet*” [18] and a support vector machine (SVM) was used for its classification. The contribution of this study has also been of great importance for the present research in the process of combining DWT with LBP to develop the splice identification algorithm.

In [19], Alahmadi et al. proposed a novel passive splicing image forgery detection scheme based on Local Binary Pattern (LBP) and Discrete Cosine Transform (DCT). First, the chrominance component of the input image is divided into overlapping blocks. Then, for each block, LBP is calculated and transformed into the frequency domain using 2D DCT. Finally, standard deviations are calculated of respective frequency coefficients of all blocks and they are used as features. For classification, a support vector machine (SVM) is used. The authors used well-known image datasets, such as Columbia, Casia v1 and v2, to benchmark their scheme. Their best results are between 96.6 and 97.5% of accuracy.

In a similar work, Ref. [20] from Alahmadi et al. proposed a novel passive image forgery detection method, based on local binary pattern (LBP) and discrete cosine transform (DCT) to detect copy–move and splicing forgeries. The schema basically extracts features, from the chrominance components of the input image, by applying 2D DCT in LBP space. For classification, a support vector machine (SVM) is used. The authors used a well-known image dataset, such as Columbia, Casia v1 and v2, to benchmark their scheme. In addition, their results are presented by distinguishing accuracy obtained on each chrominance channel, such as Cr, Cb, and CrCb. Their best results to detect the Splicing forgery inside the Casia v1 dataset are using both channels Cr and Cb with 97.5% accuracy. With the same dataset, but to detect copy-move forgery, the best results are obtained by using the Cr channel with 96.63% of accuracy. The best detection performance obtained using Casia v2 and Columbia dataset were: 97.7% with the Cr channel on Columbia dataset and 97.5% on Casia v2 using Cr and Cb channels.

Fridrich et al. in [21] proposed the first method based on the discrete cosine transform (DCT) to identify copy-paste falsification in 2003. In this work, the image is divided into overlapping blocks of 16×16, and DCT coefficients are used to extract characteristics from these blocks. After this, the DCT coefficients of the blocks are classified lexicographically. After the lexicographical classification, comparable squares are distinguished and the duplicated regions are found. In this work, robust retouching operations are performed on the image, but no other intensity test is carried out.

In 2004, Popescu et al. [22] proposed a method to identify duplicate regions of images. This method applies PCA instead of DCT coefficients, and calculates the values *Eigen* and the eigenvectors of each block of the image. Duplicate regions are automatically detected by lexicographic classification. This algorithm is an efficient and robust technique for detecting image forgery, even if the image is compressed or has a lot of noise.

Amerini et al. [23] present a technique based on the scale-invariant feature transform (SIFT) that can detect copied and pasted regions. This algorithm is also capable of detecting which geometric transformation was applied. Because the copied part looks basically the same as the original, the key points extracted in the duplicated region will be quite similar to those in the original region. The method is effective in compressed images with a low-quality factor. Forensic techniques that make use of fingerprint or sensor noise are essential to determine the origin, veracity, and nature of the data. However, they can also be used to carry out other multimedia research functions. For example, determine if one image is a copy of another by looking for matches in the digital strike of both images. Techniques based on sensor noise are mainly divided into two categories: pixel defects and sensor pattern noise (SPN). The first technique studies the defects produced in pixels, such as hot or dead pixels, row or column defects and group defects.

In [24], Luka et al. propose a method based on pixels non-uniformity (PNU), which is a great source for obtaining noise patterns. This method allows for the identification of sensors and, therefore, the camera that originated the image. Experiments were carried out with approximately 320 images taken with nine digital cameras to estimate the success rates. It is observed that the proposed method was successful in distinguishing between two cameras of the same make and model. However, the results obtained for cropped images or of different sizes were not satisfactory.

In [25], Sandoval Orozco et al. propose the mixture of two techniques (wavelet transforms combined with sensor imperfections) to obtain a better identification of sources of acquisition of images of mobile devices. The results show that sensor imperfections and wavelet transforms can jointly serve as good forensic features to help track the source camera of images produced by mobile phones. In addition, this model also allows for determining with great precision the brand and model of the device.

In [26], the authors did a comparative study to examine the effect of two state-of-the-art best texture descriptors, Multiscale Local Binary Pattern (Multi-LBP) and Multiscale Weber Law Descriptor (Multi-WLD). In its work, once the multiscale texture descriptors are extracted, from the chrominance components of the image, are passed to the Support Vector Machine (SVM) in order to identify either authentic or forged images. The performance comparison reveals that Multi-WLD performs better than Multi-LBP in detecting copy-move and splicing forgeries. Multi-WLD also outperforms state-of-the-art passive forgery detection techniques. To perform this comparison, the authors used a Casia Tide v1 dataset. The results showed a better accuracy to detect splicing and copy-move forgery by combining features extracted from the Cr and Cb channels. The best results obtained in this work showed a 94.29% accuracy to detect splicing forgery and 90,97% of accuracy to detect copy-move forgery.

Table 1 presents a summary of the detection techniques studied by comparing their results in terms of accuracy.

## 3. Digital Image Forgery

Among the types of image tampering, the following stand out: retouching, copy-paste, image splicing and digital fingerprint forgery.

### 3.1. Retouching

Image retouching is based on applying a series of filters on the original image to enhance it according to pre-defined objectives while maintaining its similar characteristics. It involves the selection of the region to be retouched and the filters to be applied to produce the most visually pleasing image [27]. This manipulation technique is commonly used in advertising, films and media [5].

**Figure 1:**
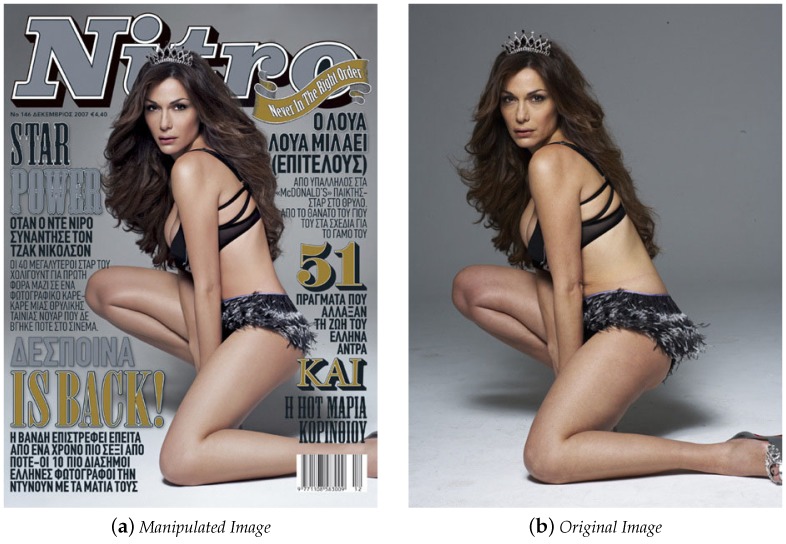
Manipulated cover of the Nitro magazine. (**a**) manipulated image; (**b**) original image.

For example, fashion magazines’ covers and advertisements usually go through some level of retouch to camouflage imperfections and thus increase the level of beauty in the photographs. Figure 1 shows the original Figure 1b and retouched Figure 1a images of a magazine cover called *Nitro*. It shows an example of photo retouching in which the model appearance was digitally altered.

### 3.2. Copy-Paste

**Figure 2:**
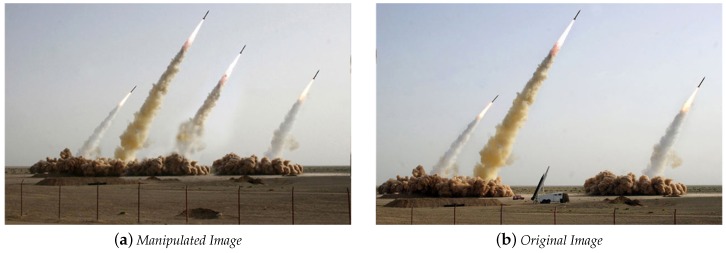
Manipulated photo of the Iranian missile launch [30]. (**a**) manipulated image; (**b**) original image.

The copy-paste technique involves copying a region in an image and pasting it over another region from the same image to conceal the image content or duplicate image regions. It can also include additional post-processing techniques such as scaling, rotating and another filters’ application. These extra procedures make the manipulation detection process more expensive. The purpose of this concealment is to hide an undesired object or increase the number of objects present in the image [28,29]. An example of this technique is shown in Figure 2a. The first image Figure 2a shows a historical photo that took place on Iran in 2008 and it depicts the successful launch of four missiles as published by Iran’s news agency (Sepah News). The original photo that was subsequently published shows that there were only three missiles launched Figure 2b. The modified image also shows the application of post-processing techniques on the smoke expelled by the missile to camouflage the manipulation.

### 3.3. Image Splicing

**Figure 3:**
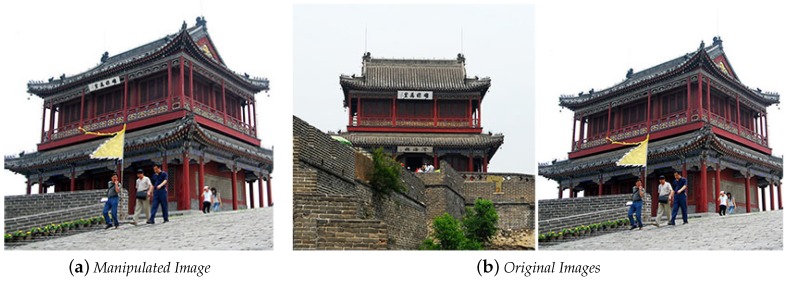
Manipulated Image [30]. (**a**) manipulated image; (**b**) original image.

Image splicing is the process of copying the region of certain image and pasting it into a different image. This technique is widely used in photomontages where two or more images are combined to give the feeling of being one image. Detection of image splicing is a complex task compared to the previous manipulation techniques due to it not being possible to look for duplicate regions since the manipulated region comes from a different image [31]. Figure 3 shows an example of the splicing technique and the two original images used in this process. From the first original image, Figure 3b, the temple sign was cropped and placed in the second original image, Figure 3b, to create the final result Figure 3a.

### 3.4. Digital Fingerprint Forgery

These techniques do not involve the image alteration itself, but the modification of the associated information (digital fingerprint) that comes from the sensor that captured the image. There are several sources of imperfections and noise introduced during the image acquisition process. Even when a uniform and fully lighted photograph is taken, small changes in intensity between individual pixels can be observed. This happens mainly for two reasons; first, there are random components such as readout noise or shot noise [32,33] and second due to pattern noise, which is a deterministic component of the sensor and it stays approximately the same if several photos of the same scene are taken. This pattern is useful to detect the source of origin of an image since each device will have a specific noise pattern [34,35].

These techniques can be divided into two types according to the objectives they pursue [35]:

- **Image anonymizing**: It refers to the destruction of image identity through the elimination of sensor noise. Image identity stands for the sensor characteristics imprinted within the image and it is of great importance because it can relate the image with the device that took the image, thus enabling identifying the owner of the device. There are several methods to remove sensor noise that goes from simple operations such as subtraction of wavelet domain characteristics to more reliable techniques such as flat fielding. Flat fielding is based on the main components of sensor noise: the fixed pattern noise (FPN) and the photo response non uniformity (PRNU). This technique allows for removing the PRNU of the image, so that the resulting image is of anonymous origin.
- **Image identity forgery**: it implies the injection of sensor noise from a different camera in an image. By modifying the source of the image, it is possible to change the real information about the model and brand of the device that produced the digital image. A widely used method to achieve identity forgery is the inverse flat fielding equation [35]. This technique requires the extraction of the PRNU and the replacement with the PRNU of a different image. Most of the anti-forensic techniques for source of origin identification use image identity to detect the mentioned forgery techniques.

## 4. Proposed Image Authentication Scheme

In this section, general considerations are presented that help to understand the proposed algorithm and the algorithm itself that accurately and effectively checks the integrity of an image is proposed.

This algorithm is based on feature extraction that combines the Wavelet transform with the histogram applied to blocks LBP and DCT.

### 4.1. General Considerations

First, it is important to know the concepts on which the algorithm used are based. In this first part of the section, these concepts will be explained.

#### 4.1.1. Color Models

The colors of an image can be represented in multiple ways. Each of these representations is called a color model. A color model assigns numerical values to different color components of an image. Among the most common are the Red–Gree–Blue (RGB), Cyan–Magenta–Yellow–Key (CMYK) and luma signal–Blue chrominance–Yellow chrominance (YCbCr) models.

- **RGB Model**: It is one of the best known due to its widespread use in computers, screens, televisions and mobile devices, among others. It is based on the representation of a color by mixing by adding the colors red, green and blue. This model assigns values between 0 and 255 to each of its three components (red, green and blue), the sum of the three will form the value that represents the color. A digital image is formed by a multitude of pixels, and each one of them will have its own value inside the model RGB being 0 the color black and 255 the color white.
- **CMYK Model**: This model is based on representing a color in the components cyan, magenta, yellow and black. This representation contrary to the previous model is done in a subtractive way and not by addition. This is why a fourth component is necessary to represent the correct black tone. The values assigned to each component range from 0 to 100, depending on the intensity wanted to the tone of the component. This model is widely used in the printing sector due to the good contrast with the one that generates the different tones of the prints. Therefore, before the printing of an image, the model RGB is usually transformed into the model CMYK.
- **YCbCr** Model: It is a color model used for the processing of digital images, this model is a nonlinear coding of space RGB and represents colors in the form of luminance and chrominance components. The luminance is represented by the symbol *Y* and the two chrominance signals are represented by *Cb* and *Cr*. *Cb* places the color on a scale between the color blue and yellow. Instead, *Cr* does it between the color red and green. Finally, the *Y* parameter offers the respective information to the black and white colors.

**Figure 4:**
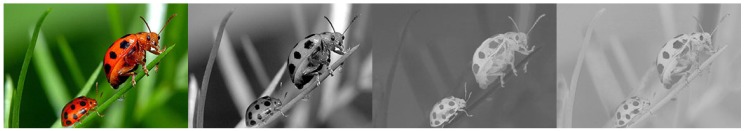
Image in the space color *YCbCr*.

Figure 4 shows an image and its components of space color *YCbCr* in the following order: luminance, blue chromatic channel and red chromatic channel.

#### 4.1.2. Local Binary Pattern

LBP is a local operator that discriminates different types of textures. When a manipulation is performed, the original texture of the image is distorted. The LBP operator defines a label called LBP code for each pixel of an image. If the value of the neighboring pixel is less than the value of the central pixel, that neighbor will be assigned the binary digit 0; otherwise, it will be assigned the binary digit 1. Once the values of the matrix are obtained, between 0 and 1, the binary number is converted into a decimal number in the sense of clockwise, in this way, the LBP code is obtained that represents the central pixel.

When LBP is used, a neighborhood value represented by the letter *P* is assigned. This value refers to the number of neighbors that surround the central pixel.

**Figure 5:**
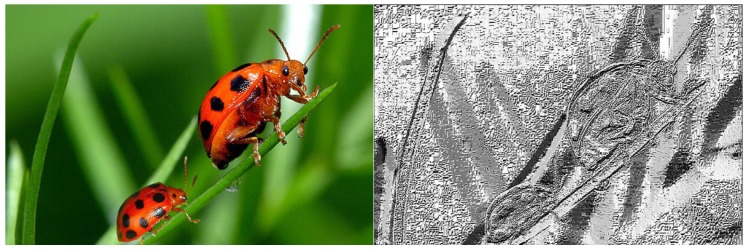
Manipulated image and its transformation applying Local Binary Pattern.

Figure 5 shows a manipulated image and its result when applying LBP.

#### 4.1.3. Discrete Cosine Transform

The discrete cosine transform DCT is a variation of the discrete Fourier transform, where the image is decomposed into sums of cosines, but using real numbers only. It only acts on periodic functions with even symmetry and the result is a sequence of real numbers. In Equation (1), the characteristic formula of the DCT-I variant is shown:

(1) $$f_{j}=\frac{1}{2}\left(x_{0}+{\left(-1\right)}^{j}x_{n-1}\right)+\sum_{k=1}^{n-2}x_{k}\cos\left[\frac{\pi }{n-1}kj\right].$$

DCT is used in the detection of manipulations and especially in compression algorithms such as JPEG for its great capacity to compact energy or most of the information in a reduced number of coefficients and for being independent of the number of input data it receives, guaranteeing a greater efficiency when working with large images.

DCT also manages to carry out the compression of images discarding the imperceptible parts for the human eye. This process uses the DCT coefficients to differentiate which points of the image present different characteristics to the rest of which are similar. This is why it is widely used in the detection of manipulations in images, and working with DCT in the chrominance channel will allow obtaining the coefficients that will indicate points of the image that stands out from the rest but which are not visible to the naked eye.

There are eight types of DCT, the most used in the compression of digital images and therefore in the field of detection of falsifications is the DCT-II (Equation (2)) whose Inverse Discrete Cosine Transform (IDCT) corresponds to the type DCT- III shown in Equation (3):

(2) $$f_{j}=\sum_{k=0}^{n-1}x_{k}\cos\left[\frac{\pi }{n}j\left(k+\frac{1}{2}\right)\right],$$

(3) $$f_{j}=\frac{1}{2}x_{0}+\sum_{k=1}^{n-1}x_{k}\cos\left[\frac{\pi }{n}\left(j+\frac{1}{2}\right)k\right].$$

#### 4.1.4. Discrete Wavelet Transform

A wavelet function is a small wave that concentrates its energy over time. It is used to analyze in terms of time and frequency stationary, non-stationary and time-variable phenomena. The wavelet transform is considered to be the most powerful form of signal representation that can be applied mainly to the processing of signals and images. There are numerous types of wavelet families, the most important are: Haar, Daubechies, Biorthogonal, Coiflets, Symlets, Morlet, Mexican Hat, Meyer, Reverse Biorthogonal, Frequency B-Spline, and Gaussian derivatives.

The discrete wavelet transform (DWT) can provide unique and discriminating representations that can quantify vital and interesting structures such as edges and details with good resolution for few coefficients. It is also computationally effective due to the small amount of data with which it works. The final wavelet coefficients can be used directly as characteristics and can be extracted directly from the wavelet domain, describing the anomalies in the image data. Basically, the discrete wavelet transform reduces the correlation between wave coefficients and provides energy compaction in some wavelet coefficients.

#### 4.1.5. Quadrature Mirror Filter

It is a filter used in signal processing that, from an input signal, divides it into two bands. The input signal is processed through two paths. In the upper path, the signal is passed through a lowpass filter and decimated by a factor of two. In the lower path, the signal is passed through a highpass filter and also decimated by a factor of two [36].

#### 4.1.6. Support Vector Machine

In machine learning SVM, it is a supervised machine learning technique used for solving pattern recognition and regression analysis problems. SVM allows complex models which are not defined simply by hyperplanes in the input space. To achieve that, the data is mapped into a higher dimension feature space. Once, in the higher dimension feature space, the SVM algorithm divides the data applying a linear hyperplane.

Since the machine learning SVM technique has proved to be an accurate and trustful prediction method [37,38,39]. In the proposed algorithm of this paper, a Kernelized SVM is used in order to classify images between authentic and manipulated.

However, the new deep learning techniques have reached excellent results in the computer vision field. The SVM technique is been used in several of the previous works related to the proposed new algorithm and, in order to compare the results from the previous works with this one, it is necessary to use SVM.

### 4.2. Contribution

In this subsection, all the performed steps by the proposed algorithm are described.

First, as input, a set of *N* images is received. Each of these M×N image are converted to the YCbCr color model. Then, the component *Y* is discarded and only the two chrominance components (Cb and Cr) are used.

As human vision notes the luminance component in a clearer way than the chrominance component, it is considered that most of the manipulation traces that cannot be detected with the naked eye can be noted in the chromatic channel. The chromatic channel describes the content of the image signal, such as edges. Thus, any inconsistency in these edges caused by the performance of common forgery techniques (copy-move or image splicing) is emphasized and, therefore, noted.

Once the image is converted, on each Cb and Cr component, the DWT is applied.

Second, each of the components Cb and Cr are then decomposed into four subbands: LL, HL, LH and HH. The discrete wavelet transform analyses an image in different scales and orientations. The splicing process often introduces a sharp transition in the two-dimensional array that represents the image in terms of edges, lines, and corners that are characterised by high-frequency components in the Fourier transform domain. These four subbands are composed by the approximation coefficient LL with the low-frequency information and three address coefficients (HL, LH, HH) with the high-frequency information in different directions (horizontal, vertical and diagonal coefficients, respectively).

Since the LL component concentrates most of the energy, the subbands HL, LH and HH are discarded by the algorithm.

Third, it is necessary to apply the QMF filter to each of the obtained LL components. The QMF filter is applied in order to get the inverse codification of the frequencies. This filter exchanges the high frequencies by the low frequencies and vicevversa.

Once the LL component is obtained and after the QMF filter, the LBP is applied.

Fourth, divide each component LL into overlapping blocks of B×B with a sliding window of one pixel and then apply LBP to each low-frequency component of the chrominance. After that, a total of (M−B+1)×(N−B+1) characteristics is obtained.

From each resulting block, the LBP is extracted using the following Equation (4):

(4) $$LBP_{p,r}=\sum_{i=1}^{p-1}s\left(p_{i}-p_{c}\right)2^{i},$$

where *p* is the number of neighbouring pixels, *r* is the radius of the neighbourhood and $p_{c}$ is the value of the central pixel. This operator calculates the LBP code by the value of eight neighbours. If the value of the neighbour’s pixel is smaller than the centre pixel, it is assigned the binary digit 0, otherwise, 1. Then, the neighbour’s binary digits are put together to build a binary code.

The local binary pattern is extracted for each block using as a neighbourhood value of 8 to obtain greater accuracy. In addition, to reduce the number of characteristics obtained with LBP, the histogram is calculated, leaving each block represented by 256 codes LBP. In this way, the changes of the different textures are observed with greater efficiency.

The local texture characteristics of the whole image can be described by means of a normalised histogram that is formed by $2^{P}$ codes LBP, where *P* is the number of neighbours that surround the central pixel. For the case of P=8, the total number of features will be 256 codes LBP for each image.

Fifth, once the histograms of all the blocks of each component are obtained, the discrete cosine transform is applied to each of them using Equation (5):

(5) $$f_{j}=\sum_{k=0}^{n-1}x_{k}\cos\left[\frac{\pi }{n}j\left(k+\frac{1}{2}\right)\right].$$

The Discrete Cosine Transform DCT has a great capacity to compact the energy or most of the information in a reduced number of coefficients and to be independent of the number of input data it receives, guaranteeing greater efficiency when working with images of large dimensions. With this, each block is represented by a set of DCT coefficients.

The result of applying the discrete cosine transform is a finite sequence of points as a result of the sum of several signals with different frequencies and amplitudes. From each set of coefficients, the standard deviation is obtained as a characteristic.

Finally, each initial chrominance (Cb and Cr) component of subband LL, from the input image, will be represented as a vector of standard deviations of the sets of coefficients DCT of all LBP blocks.The final vector of characteristics, used in the SVM, will be the concatenation of the vectors of both chrominance as shown in Equation (6).

Since the SVMs will handle a different range of values coming from each step of the algorithm. The LIBSVM framework makes a preprocessing by scaling all the values in the obtained vector in order to fit all in the same range. After that step, from the algorithms’ perspective, it doesn’t matter if features are continuous or binary, large or small.

(6) $$Image_{Final\;Feature\;Vector}=\left[Ll\;Cb_{Features\;Vector}\right]⌢\left[Ll\;Cr_{Features\;Vector}\right].$$

Once the image final feature vector is obtained and to find the best pairs of optimal classification parameters *C* and γ, the SVM use the tool *Grid-search* and *Cross-validation* from the LIBSVM implementation [40].

**Figure 6:**
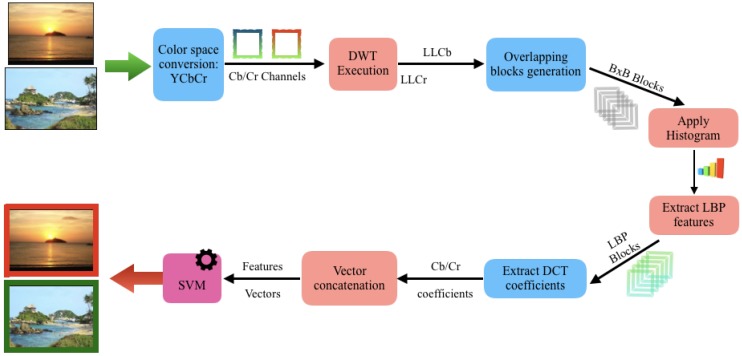
Flow diagram of the proposed system.

To have a better picture of all the steps, described above, Figure 6 shows a diagram of the feature extraction process performed by this algorithm.

## 5. Experimental Results

Throughout this section, all the experiments that have been carried out to evaluate the effectiveness of training-based manipulation identification algorithms will be shown.

### 5.1. Experiment Setup

In all the experiments carried out, *Python* has been used as a programming language, due to its great flexibility to perform data analysis and its high speed in handling input and output.

The detection of manipulations is a problem of two kinds: authentic against manipulated images. This is why, to carry out the training and classification of the data, a support machine vector has been used since it has an excellent performance when working with problems of two classes.

The library LIBSVM [40] has been used, due to its simplicity and efficiency. This implementation of SVM is today one of the most commonly used tools. As a core function, the radial base function (RBF) is used; this function is the most used in similar projects and has shown good results. The optimal values of the parameters from the core of the function RBF (γ, C) are calculated automatically by a cross-correlation process.

For the evaluation of the proposed scheme, the below public image datasets have been used to carry out the experiments using various image formats and sizes:

- CASIA v1.0 and CASIA v2.0 [15]: CASIA v1.0 dataset contains 800 authentic and 925 spliced color images of size 384x256 pixels with JPEG format. All tampered images in the CASIA v1.0 dataset are made only by splicing operation. Spliced images are generated from authentic images by crop-and-paste operation using Adobe Photoshop CS3 version 10.0.1 on Windows XP. CASIA v2.0 datset contains 7491 authentic and 5123 tampered color images. The images in CASIA v2.0 datset are with difference size, various from 240×160 to 900×600 pixels. Compared to CASIA v1.0, which only contains one image format of JPEG, CASIA v2.0 added uncompressed image samples and also considered JPEG images with different Q factors.
- Columbia [11]: Its a dataset of 1845 image blocks with a fixed size of 128×128 pixels. Develop by the DVMM Laboratory of Columbia University and CalPhotos Digital Library. It has 933 authentic and 912 spliced images.
- IFS-TC [41]: IFS-TC dataset belongs to The Image Forensics Challenge competition organized by the IEEE Information Forensics and Security Technical Committee (IFS-TC). The dataset comprises of several original images captured from different digital cameras with various scenes either indoor or outdoor. The images are divided into images “pristine” or “never manipulated” and images “forged” or “fakes”.

Table 2 shows the summary characteristics of the datasets used in this work experiments.

The characteristics of the equipment in which the experiments were carried out are presented in Table 3. It is an important factor to take into account since the execution times of the different tests vary according to the resources available.

### 5.2. Experiments

Since the proposed scheme uses an SVM as a classifier system, the SVM system was previously trained with images non-manipulated. Furthermore, using the proposed scheme, all the features were extracted from the training set of non-manipulated images and fed into the SVM.

The first set of experiments was based on verifying the variation of the precision when DCT is applied with different block sizes. In addition, in one of the mention experiments, DWT is applied before the LBP execution. The CASIA v1.0 and CASIA v2.0 datasets were used for this purpose. In Table 4, the results obtained are shown.

According to the obtained results, shown in the table above, the best accuracy is reached by using a DCT block size of 8×8. From here, all the experiments will be executed with this block size and will define the algorithm in self.

The second group of experiments is intended to define which chrominance channel shows the best results. The results obtained are shown in Table 5.

Since several previous works have compared the accuracy obtained between each chrominance channel and the combination of both, it is considered necessary to compare which channel suits the proposed algorithm with the best precision.

The obtained results have shown that the best option, for the proposed algorithm, is to use the Cb channel with an Average Accuracy of 98.10%. Given that the Cb channel is the one that reaches the major precision over all the used datasets.

Comparing both previous results, Table 6 shows the best results on each case with the above-defined block size.

The third set of experiments was based on verifying the variation of the precision when applying the Quadrature Mirror Filter (QMF) on the manipulation identification algorithm based on DWT.

The test was carried out applying LBP on the Wavelet coefficients in a direct way and another passing the wavelet coefficients by QMF before applying LBP.

The CASIA v1.0 and CASIA v2.0 datasets were used for this purpose. Table 7 shows the results obtained.

As can be seen in the table, the algorithm that uses QMF achieves a slight increase in accuracy in both data sets.

The fourth group of experiments was the execution of the proposed algorithm with all the acquired tweaks that increase the precision and the efficiency. The results obtained are shown in Table 8.

As can be seen in the table, the algorithm based on DWT obtains better results in the three data sets and is faster than the algorithm based only on DCT. This is due to the overlapping blocks division that it performs. As a reference, the execution time of both algorithms was measured for an image with dimensions 1280×854. The algorithm based on DCT had an execution time of 89 s, the algorithm based on DWT being more efficient with a run time of 16 seconds. Likewise, it was observed that the larger the image size, the algorithm based on DCT considerably increases the processing time of the images. However, in the algorithm based on DWT, there is a slight increase in the execution time.

Finally, both algorithms get good results with compressed images, such as images with JPEG format. Good results were also obtained for images with PNG format. The two algorithms cannot reach a precision higher than 50% with images in TIFF format.

Before a general comparison of the proposed algorithm with others in the literature, the results obtained from the test carried out over the different chrominance channels are compared with some other authors, with results of its algorithms being presented with separation of each chrominance channels. Table 9 shows the obtained results from the proposed algorithm and the results from previous works.

As the table shows, the proposed algorithm performs a better detection in all the tested datasets with all the chrominance channels, compared with the state-of-the-art algorithms.

Table 10 presents a comparison between the proposed authentication technique and state-of-the-art forgery detection methods, which use the same datasets and SVM classifier with RBF kernel. It can be observed that the proposed technique outperforms the existing techniques.

## 6. Conclusions and Future Work

Digital images contain a large amount of relevant information. Because of this, they have become very important in legal procedures as they can be used as evidence of great value in a trial’ resolution. For the images to be valid as evidence, it must be possible to guarantee its authenticity and integrity in a reliable way. Nowadays, there are numerous image editing applications that deliver highly professional results, thus making it harder to detect forgeries. Therefore, it is of great interest to have forensic tools for image forgeries detection that can guarantee the image integrity.

In this work, an exhaustive study on existing forgery detection techniques has been carried out, putting emphasis on image splicing and copy-paste detection. In addition, a new approach for forgery detection that improves the results obtained in previous investigations was presented. This technique is based on the DWT with LBP histograms and QMF.

This technique extracts the wavelet characteristics and obtains the histogram by applying LBP on the image under analysis. Finally, it improves the detection accuracy with QMF and classifies the image.

A set of experiments have been carried out using four benchmark datasets (CASIA v1.0, CASIA v2.0, Columbia and IFS-TC datasets) of the state of the art to evaluate the proposed technique. The results, after carrying out all of the experiments, shows that the proposed algorithm reaches a better accuracy compared with the state-of-the-art algorithms using the same datasets, showing a better accuracy on tampering detection in most of the cases.

At the same time, the algorithm proved to be efficient in tests made after comparing their execution time with other algorithms. This is due to the algorithm not dividing the image into blocks, thus working directly with the original image.

Since the deep-learning approach arises as a solution for computer vision problems [42,43], as future work, its necessary to execute and compare this algorithm using deep-learning techniques and increasing the size of the used datasets.

## Figures and Tables

**Table 1:** Main aspects of related previous works.

Approach	Extracted	Classification	Dataset	Accuracy
	Features	Method		
[5]	Modelling	SVM	Web Sites	98.75%
	geometric changes			
[6]	Facial features	Neural Network and SVM	ND-IIITD	87%
			retouched faces	
[7]	Facial features	SVM	YMU, MIW	93%
[9]	Facial features	SVM	YMU, MIW	98.5%
[10]	MBDCT	SVM	Columbia	91.40%
[12]	MBDCT, IQM	SVM	Columbia	87.10%
[13]	LBP, MBDCT, PCA	SVM	Columbia	89.93%
[14]	Chromatic Channels,	SVM	CASIA v2.0	95.6%
	Markov chain			
[16]	Chrominance channels,	SVM	CASIA v1.0	94.7%
	RLRN vectors			
[17]	LBP, DWT	SVM	LiveSet	92%
[20]	LBP, DCT	SVM	CASIA v2.0	97.50–97.77%

**Table 2:** Features of the used datasets.

Datasets	Format	Resolution	Number of Images		
			Original	Fake	Total
CASIA v1.0 [15]	JPEG	384 × 256	800	921	1721
CASIA v2.0 [15]	JPEG, BMP,	240 × 160 to	7491	5123	12,614
	TIFF	900 × 600			
Columbia [11]	TIFF	757 × 568 to	183	180	363
		1152 × 768			
IFS-TC [41]	PNG	1024 × 768 to	424	451	875
		3648 × 2736			

**Table 3:** Features of the experimentation equipment.

Resources	Features
Operating System	Ubuntu 17.04
Memory	12 GB
Processor	Intel^®^ Core^™^ i5-6200U CPU 2.30GHz x 4
Graphic Card	Intel^®^ HD Graphics 520 (Skylake GT2)
HDD	64 GB

**Table 4:** Variation of precision using DCT with different block size 8×8 and 16×16.

Dataset	DWT-LBP-DCT		LBP-DCT	
	Block Size 8×8	Block Size 16×16	Block Size 8×8	Block Size 16×16
CASIA v1.0	77.86%	69.27%	85.71%	76.12%
CASIA v2.0	65.11%	65.83%	97.12%	74.10%

**Table 5:** Algorithm executed individually on each component Cr and Cb and also combined.

Dataset	Cb	Cb + Cr	Cr
CASIA v1.0	98.57%	98.22%	99.64%
CASIA v2.0	99.50%	99.64%	99.64%
IFS-TC	96.19%	93.57%	93.57%
**Average**	98.10%	97.14%	97.62%

**Table 6:** Comparison of the best accuracy of LBP-DCT with DWT-LBP-DCT only with Cb channel.

Dataset	DCT Block Size 8×8	
	DWT-LBP-DCT	LBP-DCT
CASIA v1.0	85.71%	98.57%
CASIA v2.0	97.12%	99.50%

**Table 7:** Variation of the precisions when applying Quadrature Mirror Filter.

Dataset	DWT-LBP-DCT	DWT-QMF-LBP-DCT
CASIA v1.0	97.66%	98.57%
CASIA v2.0	98.73%	99.50%

**Table 8:** Accuracy obtained by both algorithms.

Dataset	DWT-QMF-LBP-DCT	LBP-HIST-DCT
CASIA v1.0	98.57%	53.72%
CASIA v2.0	99.50%	94.94%
IFS-TC	96.19%	70.22%

**Table 9:** Chrominance channels’ comparison.

Method	Cr		Cb		CbCr	
	CASIA v1.0	CASIA v2.0	CASIA v1.0	CASIA v2.0	CASIA v1.0	CASIA v2.0
Proposed Method	99.64%	99.64%	98.57%	99.50%	98.22%	99.64%
[20]	96.52%	97.41%	96.19%	97.50%	96.90%	97.50%
[19]	95.80%	95.80%	96.50%	96.50%	97%	97.50%
[26]	92.62%	–	88.66%	–	94.19%	–

**Table 10:** Comparison between the accuracies (%) of the proposed technique and state-of-the-art approaches.

Approach	Accuracy (%)			
	CASIA v1.0	CASIA v2.0	Columbia	IFS-TC
Proposed Algorithm	98.57%	99.50%	93.89%	96.19%
[10]			91.40%	
[12]			87.10%	
[13]			89.93%	
[14]		95.6%		
[16]	94.7%			
[20]		97.50–97.77%		

## Abbreviations

The following abbreviations are used in this manuscript:

BMP	Windows Bitmap
CMYK	Cyan, Magenta, Yellow, Key Color Model
DCT	Discrete Cosine Transform
DWT	Discrete Wavelet Transform
FPN	Fixed Pattern Noise
HDD	Hard Disk Drive
IDCT	Inverse Discrete Cosine Transform
IQM	Image Quality Metrics
JPEG	Joint Photographic Experts Group
LBP	Local Binary Pattern
MBDCT	Multi-size Block Discrete Cosine Transform
PCA	Principal Component Analysis
PNG	Portable Network Graphics
PRNU	Photo Response Non Uniformity
QMF	Quadrature Mirror Filter
RBF	Radial Basis Function
RGB	Red Green Blue Color Model
RLRN	Run-Length Run-Number
SVM	Support Vector Machine
TIFF	Tagged Image File Format
YCbCr	Luma signal, Blue Chrominance, Yellow Chrominance Color Model
